# National policy development for cotrimoxazole prophylaxis in Malawi, Uganda and Zambia: the relationship between Context, Evidence and Links

**DOI:** 10.1186/1478-4505-9-S1-S6

**Published:** 2011-06-16

**Authors:** Eleanor Hutchinson, Justin Parkhurst, Sam Phiri, Di M Gibb, Nathaniel Chishinga, Benson Droti, Susan Hoskins

**Affiliations:** 1 Department of Global Health and Development, London School of Hygiene and Tropical Medicine, 15-17 Tavistock Place, London, UK; 2 The Lighthouse Trust, Mzimba Rd, Kamuzu Central Hospital, P.O Box 106, Lilongwe, Malawi; 3 Medical Research Council, Clinical Trials Unit, 222 Euston Road, London, UK; 4 Zambia AIDS-Related TB Project, Ridgeway Campus, School of Medicine PO Box 50697, Ridgeway, Lusaka 1010, Zambia; 5 Medical Research Council Programme on AIDS, Uganda Virus Research Institute, P.O. Box 49, Entebbe, Uganda

## Abstract

**Background:**

Several frameworks have been constructed to analyse the factors which influence and shape the uptake of evidence into policy processes in resource poor settings, yet empirical analyses of health policy making in these settings are relatively rare. National policy making for cotrimoxazole (trimethoprim-sulfamethoxazole) preventive therapy in developing countries offers a pertinent case for the application of a policy analysis lens. The provision of cotrimoxazole as a prophylaxis is an inexpensive and highly efficacious preventative intervention in HIV infected individuals, reducing both morbidity and mortality among adults and children with HIV/AIDS, yet evidence suggests that it has not been quickly or evenly scaled-up in resource poor settings.

**Methods:**

Comparative analysis was conducted in Malawi, Uganda and Zambia, using the case study approach. We applied the ‘RAPID’ framework developed by the Overseas Development Institute (ODI), and conducted a total of 47 in-depth interviews across the three countries to examine the influence of context (including the influence of donor agencies), evidence (both local and international), and the links between researcher, policy makers and those seeking to influence the policy process.

**Results:**

Each area of analysis was found to have an influence on the creation of national policy on cotrimoxazole preventive therapy (CPT) in all three countries. In relation to context, the following were found to be influential: government structures and their focus, donor interest and involvement, healthcare infrastructure and other uses of cotrimoxazole and related drugs in the country. In terms of the nature of the evidence, we found that how policy makers perceived the strength of evidence behind international recommendations was crucial (if evidence was considered weak then the recommendations were rejected). Further, local operational research results seem to have been taken up more quickly, while randomised controlled trials (the gold standard of clinical research) was not necessarily translated into policy so swiftly. Finally the links between different research and policy actors were of critical importance, with overlaps between researcher and policy maker networks crucial to facilitate knowledge transfer. Within these networks, in each country the policy development process relied on a powerful policy entrepreneur who helped get cotrimoxazole preventive therapy onto the policy agenda.

**Conclusions:**

This analysis underscores the importance of considering national level variables in the explanation of the uptake of evidence into national policy settings, and recognising how local policy makers interpret international evidence. Local priorities, the ways in which evidence was interpreted, and the nature of the links between policy makers and researchers could either drive or stall the policy process. Developing the understanding of these processes enables the explanation of the use (or non-use) of evidence in policy making, and potentially may help to shape future strategies to bridge the research-policy gaps and ultimately improve the uptake of evidence in decision making.

## Background

The creation of health policy is a political and social process, in which local context can impact on the ways in which evidence makes its way into national policy [1]. Yet rigorous policy analyses in resource poor settings are rare, despite frameworks which have been constructed to help analyse and understand the various influences that shape policy development in these contexts [2,3]. A recent overview of the health policy literature in low and middle income countries, concluded with a call for analyses which consist of comparative, multi-country studies; utilising rigorous case studies which deliberately seek to explain health policy change in these settings [4].

National policy making around cotrimoxazole (trimethoprim-sulfamethoxazole) preventive therapy (CPT) in developing countries offers a particularly useful case for the application of a policy analysis lens to issues in health policy development. The provision of cotrimoxazole as a prophylaxis is an inexpensive and highly efficacious preventative intervention in HIV infected individuals. In Africa it works by reducing bacterial infection, malaria, and isosporiasis; reducing morbidity (by 43%) and hospital admission (by 23%) among children with HIV/AIDS and reducing mortality (by 31%) and morbidity (by 27%) among adults with HIV/AIDS [5,6]. Research demonstrating its efficacy in sub-Saharan Africa was first published in 1999, and since then a number of studies in adults and one in children have been published, providing a solid evidence base demonstrating its efficacy, cost effectiveness and feasibility of implementation [5,7-20]. Yet, the policy response to CPT has varied across Africa, with some countries not taking CPT into policy at all, and others varying as to when it should be prescribed (such as at different CD4 cell count levels and/or different disease stages of patients [21]). Amongst health researchers, frustration at the slow scale-up is evident: three separate papers in leading health journals have sought to directly address the slow scale-up of CPT in resource poor settings [21-23]. None, however, addressed the issue of policy development in detail.

In order to understand the differential processes by which CPT evidence has been taken up into national policy in Africa, we present a report of comparative analysis (case studies) of three African countries: Zambia, Malawi and Uganda. These countries lend themselves to useful comparison for analysis. All are resource poor with generalised HIV epidemics, limited health service infrastructure and with high levels of bacterial resistance to cotrimoxazole. All conducted research on the efficacy of CPT between 1999 and 2003 – although the research used different designs (Zambia has provided the only randomised controlled trials (RCT) on the efficacy of CPT in sub Saharan Africa since 2000). In policy terms, however, they responded very differently to the body of evidence on CPT.

This paper provides an analysis of the differing country policy processes, and provides a reflection and theorisation on ways in which context, evidence and networks of researchers and policymakers influence policy development. The paper explains how the developing country context influences the formation of local evidence, the take up of international evidence, and the shape and form of the networks of policy actors influential in these processes. It also explains the roles that actors play to get research into policy and how these roles depend on the types of barriers faced – barriers which themselves arise from the national policy context and the nature or understanding of the evidence itself.

## Methods

**Figure 1:**
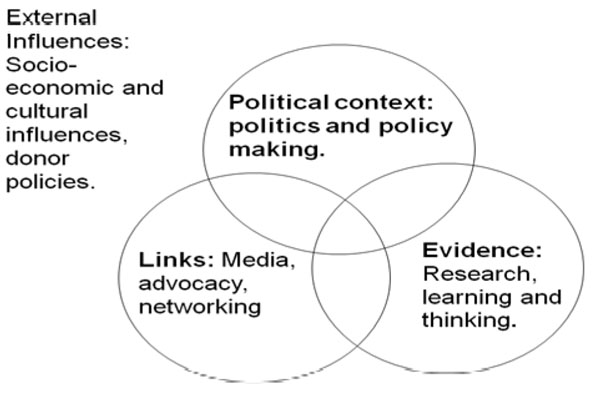
The ODI RAPID Framework

To explore the factors influential in the research-to-policy process for CPT in the three low income countries, we utilised the ‘RAPID’ (Research and Policy In Development) framework developed by the Overseas Development Institute (ODI) (see figure 1) [3,24] Constructed in the recognition that policy making is a multi-dimensional process, the RAPID framework has been applied to the analysis of policy-making in a variety of sectors in developing countries [25-27]. This framework is particularly useful for the purposes of our research, identifying research evidence as just one of four elements which shape policy development in developing countries [28].

Comparative analysis was conducted in Malawi, Uganda and Zambia. Background research identified the international evidence base for CPT, recommendations made by key policy influencing bodies (the WHO, UNAIDS, and UNICEF), and the policies on CPT in each country. In-depth country case studies were conducted. Published and unpublished documentation was supplemented with in-depth interviews with 47 stakeholders involved either centrally or peripherally in the development of the cotrimoxazole policy (15 in Malawi, 15 in Uganda and 17 in Zambia). These stakeholders were drawn from government, national and international non-governmental organisations, multilateral agencies, research institutions and hospitals. The sampling was both purposive and used snowball techniques. We developed an interview guide in English which followed themes of the RAPID framework. This guide was divided into four subject areas process, context, evidence and links. The first allowed for an overall description of the research to policy process and the three subsequent areas for discussion were used to gain data on the ways in which the process was structured by these factors. All Interviews were recorded and transcribed by the lead researcher (EH). The analysis utilised the Nvivo qualitative software analysis package, with highest level data coding following the three main areas of analysis: context, evidence and links. A three page document detailing the main findings of the country case studies was produced for each country. All informants had the opportunity to read this document and comment on the findings from their own country study: modifications and clarifications to the research findings were made accordingly.

At the beginning of each interview, research aims were described and informed consent was secured. All efforts to render the participants anonymous in any papers published have been made, but due to the nature of policy making (small numbers of often high profile participants), it was explained that it was not possible to provide complete anonymity. Key informants had the opportunity to state that they did not want to quoted, even anonymously. The key informants are identified in the text only by the country study which they belong to, their anonymous informant number, and the date on which they were interviewed.

## Results

### International research and recommendations

In the 1990s, two clinical trials on the efficacy of CPT were undertaken in Cote d’Ivoire [7,8]. The results showed that CPT was effective at an early stage of HIV disease: reducing morbidity and mortality in HIV infected TB patients, and reducing severe clinical events in HIV infected adults [7,8]. Following the release of these results, the WHO and UNAIDS released provisional recommendations that CPT be given to all HIV infected adults with a CD4 count of 500 cells/mm3 or less, or with WHO clinical stage 2-4, to all infants HIV-infected or HIV-exposed; and to all children over the age of 15 months with symptomatic HIV disease [29]. These recommendations were somewhat controversial, as many researchers in other country settings were not sure of the generalisability of the research findings across Africa.

The publication of the provisional recommendations, however, resulted in the halting or redesign of three other placebo-controlled trials underway on CPT in Malawi, Senegal and South Africa [11,15,30]. At national level in many African countries the provisional recommendations were considered to be based on insufficient evidence to justify their implementation. This was primarily as the Cote d’Ivoire studies were conducted in an area of low bacterial resistance to cotrimoxazole, while much of southern Africa is known to have high bacterial resistance to the drug [21]. Over the following eight years, a further nine major studies were conducted into the efficacy of CPT for HIV related illness, including specific studies for HIV infected patients with tuberculosis (TB) [5,9,12,13,15-17,31,32]. All but one of these studies were conducted in areas of high bacterial resistance to cotrimoxazole (the remaining one, based in South Africa [9], did not state levels of resistance) and all but one study [31] reported significant impacts on either mortality, morbidity or both among HIV infected patients.

### National policy responses

Additional file 1 shows the key research and policy events at both international and national level for each country. Of the three, Malawi was the first to begin research on CPT, focusing on its use for HIV infected TB patients. Funding for the first Malawian trial was halted following the publication of research results in Cote d’Ivoire. It was re-designed without its control arm to examine the efficacy of two different doses of CPT and compare these to historical data [11]. This was one of five efficacy or feasibility studies which were either complete or underway by 2002, when the Malawi government agreed to a phased scale-up of CPT for HIV infected TB patients (both before and after treatment for TB) [15,17,33-35].

In Uganda, interviewees reported that the government initially rejected the request by the United States Centers for Disease Control and Prevention based in Uganda (CDC/Uganda) and the local NGO The AIDS Support Organisation (TASO) to scale up CPT in TASO clinics, requesting instead data on the efficacy of CPT in Uganda (an area of high bacterial resistance) (UKI 2 16/12/2009; UKI 9 20/01/2010). Two observational studies were subsequently conducted [13,16], and in 2003 the Ministry of Health agreed that CPT could be scaled up by CDC/Uganda, the UK Medical Research Council Uganda Virus Research Institute (MRC UVRI) and TASO. At the time, the government rejected the *national* scale-up of CPT as they had outstanding questions about cost and the best way of reducing opportunistic infections among HIV infected patients (such as investment in safe water supply) (UKI 7 28/01/2010). Following two years of lobbying and activity by CDC/Uganda, during which time further evidence on the efficacy of CPT in areas of high bacterial resistance emerged, the government agreed in 2005 to a broad national policy on the scale up of CPT for all HIV infect adults and HIV infected and exposed children [5].

The same year (2005), primarily on the basis of the research from Uganda and Zambia, the Malawian HIV Programme adopted a broader policy, providing CPT for HIV infected adults and children with and without TB. In Zambia an RCT on the efficacy of CPT among HIV infected children released research results in 2003 (the results of the RCT on the efficacy of CPT among HIV positive TB patients were not published until 2008 and the results of the third RCT on the efficacy of CPT among HIV positive post-partum women have not yet been released [32]). The results of the paediatric study showed that CPT was highly effective at reducing morbidity and mortality despite high levels of bacterial resistance to cotrimoxazole [5]. Although these findings, published in 2004, came from within Zambia, scale-up of CPT was not undertaken by the National AIDS Council on the basis that there was no sufficient infrastructure or funding to ensure its provision. It was not until 2006 that the government agreed to a policy of providing CPT for HIV related illness. The following year detailed guidelines were published.

In addition to these different timelines to policy development, the content of national CPT policies also had some differences between our three case study countries. Additional file 2 summarises and compares the content of the three countries’ CPT policies.

### Explaining uptake and use of research in policy

Despite similarities in terms of level of economic development, burden of HIV and common concerns with bacterial resistance, the three countries illustrate different pathways from evidence to policy on CPT, and differences in policy content. We explored these research-to-policy processes explicitly through the use of the RAPID framework to identify the key factors at play, and how they interact to shape the uptake of research evidence in low-income countries.

### Context

The first area to be explored was the health policy context. This was taken to broadly include the institutional structures in place through which policy is made in each settings, as well as the political agendas and issues facing policy makers in HIV. In our comparison, four main areas were most important in shaping the development of CPT policy:

• Government structures and their focus

• Donor interest and involvement

• Healthcare infrastructure

• Other uses of cotrimoxazole and related drugs in the country

The focus of the existing government organizations was critically important in all countries. In 2002, Malawian national HIV programming was focused on prevention. Lacking a bio-medical approach, it left staff relatively disinterested in the potential that CPT had to reduce HIV related infections (MKI 13 6/11/2009, MKI 6 16/2/2009, MKI 8 17/02/2009). In marked contrast, the Malawian National Tuberculosis Control Programme was actively seeking bio-medical interventions that could reduce the high numbers of deaths among HIV infected TB patients, which led to CPT being seen as an attractive option by this body (MKI 13 12/11/2009, MKI 9, 11/02/2009, MKI 6 16/02/2009, MKI 2 22/2/2009). According to one policy maker:

“The TB programme was seeing a lot of mortality in TB and HIV patients and we did not have anything to give these patients. So you find that the TB programme was strong, it was mature and well organised, but we had not much to offer directly to patients who were co-infected and this was the starting point for the programme.” (MKI 13 12/11/2009)

However, interest in pursuing a bio-medical approach to HIV did not guarantee interest in CPT in all settings. In Zambia, interviewees reported a biomedical focus, but within this an emphasis on anti-retrovirals (ARVs) at the exclusion of CPT over CPT (ZKI 3, 26/09/2008). Interest in ARVs in Zambia was politically charged, with the explicit involvement of the President of Zambia in seeking nationwide access through the public sector.

“At the time that we started with the ARV programme the donors strongly felt that Africa did not have the capacity to handle ARVs, so the first ARVs that were bought in Zambia were bought by the Zambian government. The donors provided kits for testing for HIV but they did not think that we should have an ARV programme.” (ZKI 8 10/11/2009)

According to one informant, this heightened focus on ARVs simply meant that policy makers were less interested in CPT, drawing their attention away from the important research results of the efficacy of CPT (ZKI 3, 26/09/08). According to another informant, the emphasis on ARVs at the exclusion of CPT was considered to be because it was difficult to scale up both ARVs and CPT at the same time (ZKI 8 10/11/2009).

Of all of the countries in our study, the development of CPT policy in Uganda was apparently the most explicitly influenced by a donor organisation. In 1999 CDC/Uganda had funds for programmatic work and were looking for low cost interventions to prolong the lives of those with HIV (UKI 9 29/01/2010). The CDC had been involved in the research on CPT in Cote d’Ivoire, and senior personnel in the Ugandan CDC office were keen to fund the implementation of CPT within clinics being run by the Ugandan NGO, TASO (UKI 2, 16/12/2009; UKI 11, 8/02/2010). CDC/Uganda had access to funding for CPT; expertise and funding to conduct a study of its efficacy (when evidence was requested by the government); and the means to support the policy development process. It was perceived to be active throughout the policy development process (UKI 2, 16/12/2009, UKI 3 17/12/2009, UKI 9, 29/01/2010, UKI 10, 11/02/2010, UKI 14, 15/03/2010). According to one researcher:

The Ugandan government were uncomfortable with the international data so the CDC conducted the Ugandan study on the use of cotrimoxazole. As soon as they completed this study, they approached the ministry of health again…..As soon as the MOH gave [the CDC] the go ahead, gave the technical teams the go ahead to develop their policy, [the CDC] worked with the technical staff at the Ministry of Health to draft the initial draft of the policy guidelines.... The CDC has the advantage of being able to support policy development but also has additional resources to help the implementation of policy. For cotrimoxazole, the amount of money that was being put aside for the cotrimoxazole, the CDC agreed with the MOH that it would help and it committed substantial resources in terms of the amount of cotrimoxazole required. (KI 14, 15/ 03/2010)

In Malawi and Zambia, donor agencies were not seen as having such a direct role in the creation of local research or shaping of policy, despite being involved in providing funds for the research in both countries, either directly (through the UK’s Department for International Development Zambian office for the Zambian trials) or through national programming (a variety of donor agencies through the TB Programme in Malawi).

In all countries, the structural and economic feasibility (i.e. having a healthcare infrastructure and sufficient funds) of implementing CPT was a further concern at the point at which national policy was considered and either developed or rejected. Within Uganda and Malawi, the partial scale-up of CPT was expected to (and did) occur within well-established health programmes (TASO and the National TB Control Programme, respectively), in which clinics were already providing care to HIV infected patients. CPT could be added to an already functioning structure providing care to HIV infected patients, and in both cases funds were known to be available to purchase cotrimoxazole. In Zambia, policy makers had been concerned about the lack of infrastructure through which CPT could be provided to patients, and this was considered to be a major obstacle to policy creation (ZKI 13, 23/10/2008, ZKI 15 28/10/09). One medical doctor involved in the discussion of cotrimoxazole at national level argued:

[the concern was] whether it was feasible to [implement cotrimoxazole], it was the cost - can we afford to do this - and how will we do it. At the time we had ARVs in three pilot sites, so we could accompany the ARVs with cotrimoxazole but we weren’t everywhere…..people were thinking that this is good, this is something that we could do but how. So, it depended between the “how” for all of these years. (ZKI 13 23/10/2008)

The fact that policy makers were willing to consider the development of infrastructure to provide ARVs means that lack of infrastructure is not an insurmountable problem (structures can be developed). The combination of lack of infrastructure with the relatively low perceived priority of CPT (compared with ARVs), seems to have created a context in Zambia in which CPT was not able to be adopted into policy as early as in the comparison countries. Only later, when ARVs had been scaled up and with the necessary healthcare infrastructure in place, was it seen as feasible to adopt CPT in Zambia.

The final point about the healthcare contexts relates to concerns raised about the potential for CPT to increase resistance to cotrimoxazole and related drugs. These concerns were raised in both Malawi and Uganda (but not Zambia). In those countries sulfadoxine/pyrimethamine (SP) (which belongs to the same class of antibiotics as cotrimoxazole) was used as (part of) the first line for malaria treatment. Concerns were raised by those involved in malaria programming and research in both countries that the widespread use of CPT might render SP ineffective (MKI 13, 12/11/2009, MKI 12, 18/02/2009, MKI 10, 20/2/2009, MKI 6, 16/02/2009, MKI 5, 15/02/2009, MKI 4 29/01/2009, MKI 2, 27/02/2009). Cotrimoxazole was also used to treat respiratory infections in both countries, although the use of cotrimoxazole for this purpose was highlighted more by Ugandan rather than Malawian informants (UKI 8, 29/01/2010, UKI 2, 16/12/2009, UKI 3, 17/12/2009). However, by the time the 2005 policy was created in both Malawi and Uganda, both countries had changed their first line treatment for uncomplicated malaria away from SP and as such concerns about cross resistance were less relevant.

### Evidence

In all three countries studied, the 2000 WHO/UNAIDS provisional recommendations were considered to be based on insufficient evidence to justify implementation. This was primarily because Cote d’Ivoire is an area of low bacterial resistance to cotrimoxazole. In Uganda, the Government was also concerned that widespread use of CPT would create further resistance to cotrimoxazole (rendering it useless as a treatment), and in Malawi the government considered the spectrum of diseases in HIV infected patients to be different to those in Cote d’Ivoire (UKI 9 29/01/2010; MKI 7 17/02/2009). Following the publication of the 2000 WHO/UNAIDS recommendations, in both Malawi and Uganda the government considered that further trials would no longer be considered ethical. This left the countries in the difficult position of having insufficient evidence to warrant implementation but unable to undertake experimental trials. Criticism of the publication of the 2000 WHO/UNAIDS recommendations was most outspoken in Malawi, where UNAIDS funding for a randomised trial measuring the efficacy of CPT in reducing mortality and morbidity among HIV positive patients with active tuberculosis was suspended [36]. Two Malawian informants argued that the publication of the 2000 recommendations had been counter-effective, slowing the provision of CPT across the region (MKI 2, 27/02/2009, MKI 7 17/02/2009). One researcher argued:

If you look at the letter written to the Lancet, it said that this was completely the wrong decision of UNAIDS [to publish the 2000 guidelines], because there were studies going on in Malawi, Cape Town, and Senegal. All three of those studies stopped. The next RCT was Chintu [in 2004]. In retrospect, I think that UNAIDS held up the implementation of CPT in Africa. (MKI 2 27/02/2009)

In contrast, in Zambia, after careful consideration the government allowed three clinical trials to go ahead [30].

The model of research conducted in each country was therefore different. In Zambia, three experimental trials were conducted (among HIV infected children, post partum women and TB patients) and no specific operational research was undertaken [5,32]. In Malawi, three pieces of operational research were conducted on the effectiveness of CPT in HIV infected TB patients, with the specific intent of informing policy and improving the outcome of patients (MKI 12 18/03/2009). Similarly in Uganda, two pieces of operational research were conducted, examining efficacy, and addressing issues which had been raised by the Ministry of Health: cost per person, and the development of cotrimoxazole resistance pathogens in diarrhoea and SP resistant malaria.

It was, therefore, the less epidemiologically robust research conducted in Uganda and Malawi that appears to have been more easily translated into practice. In both countries the policy relevance of the operational research was established as the study was being designed, and both studies paid attention to the feasibility of implementation (a key concern raised in terms of context, see above). According to one researcher:

In the beginning there was a list of questions that the Ministry [of Health] raised: is [CPT] going to work or not, will [CPT] increase resistance, is [CPT] going to be too costly for us? So we embedded a cost effectiveness plan from the very beginning and we made sure that we spent the time answering those other questions. We showed that it was effective, we showed that it was cost effective, in fact it was cost saving, we looked to see whether it increased resistance in malaria and saw that it actually decreased the resistance to Fansidar. We treated those questions seriously, we answered them scientifically, effectively and they happened to be answered in support of the provision of cotrimoxazole. (UKI9 21/01/2010)

In Zambia, while the experimental trials clearly showed the efficacy of CPT in an area of high bacterial resistance, no data regarding the feasibility of implementation was provided at that time. One senior policy maker argued:

In principle people agreed [with the research results] but then you need to quantify and buy the cotrimoxazole. These things were never worked out, you know the CHAP study does not give those parameters so those were the things that were not worked out and we needed to have the funding available to procure the cotrimoxazole if it was going to be universally applied. Those were the challenges. (ZKI 15 28/10/09)

Further, the results of the paediatric CHAP (cotrimoxazole as prophylaxis against opportunistic infections in HIV-infected Zambian children) trial were interpreted by senior clinicians and policy makers as being of importance for clinical decision making, but not necessarily requiring a new national policy (ZKI 8, 10/10/09, ZKI 15 28/10/09). The Zambian research results were central in the development of the national policy in Malawi in 2005 and important in the development of policy in Uganda in the same year. It took a further two years, however, before the national Zambian policies and guidelines were published.

### Links

The links element of the framework focused on the importance of key actors and their interactions to explain how research evidence can be taken up into policy. In particular, it was found important to understand the roles of policy networks, policy entrepreneurs (individuals crucial to the development of the policy who bridge other influential groups) and policy champions (groups of experts and policy makers providing support to the policy entrepreneur).

Unsurprisingly, in each of the three countries, two important networks were identified: researchers, who were seen to be experts in the field; and policy makers – the key actors who would be making decisions on CPT. A third network of NGOs and implementing actors was also identified by some respondents.

It was found that each successful policy process appeared to be reliant on a policy entrepreneur, particularly when there were other elements which posed barriers to formation of a CPT policy (doubt over the evidence relevance, or concerns of the impact of CPT on other treatments). These entrepreneurs were able to link between the research and policy networks, and also to rally support for CPT when policy making apparently stalled. In Zambia in 2004, and in the Malawi HIV Programme in 2002 there was no policy entrepreneur identified who had been working for such changes (in Malawi the entrepreneur was in the TB Programme).

Strikingly, the policy entrepreneurs in all three countries had similar backgrounds. They were physicians either currently or previously involved in medical research whom, while the policy was being developed, were in senior policy positions in well funded organizations/programmes. They were, however, placed in different institutions: with one in government (Malawi), one at an NGO (Zambia) and one in a donor agency (Uganda). One researcher in Malawi considered that this institutional position of the policy entrepreneur was also significant, allowing the government to have ownership of the cotrimoxazole research. He argued:

It was very different when someone from the World Health Organisation or someone from an academic institution goes to the MOH and says “Here is a study here are the results and would you like to implement it?” In the case of cotrimoxazole there was someone from the Ministry of Health in a high position, very well respected, a good researcher, and a good programme person. He said, “Here are the results, we have been involved with several partners, you have heard of this throughout.” And the Ministry says “Yes, sure this is part of what we have been involved with.” (MKI 12 18/03/2009)

The visibility of the entrepreneurs was variable: in Malawi the policy entrepreneur was identified by 11 out of 15 informants (including the policy entrepreneur himself). In Zambia, the policy entrepreneur was identified by 3 out of 15 (including the policy entrepreneur); and in Uganda, the policy entrepreneur was identified by 5 out of 15 informants (including the policy entrepreneur). In Malawi and Uganda, the policy entrepreneurs were both involved in local research conducted while in Zambia, the policy entrepreneur utilized the results of research already undertaken to support his case for a national CPT policy.

The policy entrepreneurs did not work alone, however. In each country they were surrounded by a supportive group of influential actors termed here ‘policy champions’. These were researchers and/or policy makers, who provided support and expertise, helped to drive the policy forward and who recruited support from other physicians, policy makers and researchers. The entrepreneurs may have been the ones who identified strategic points to press for change, and who linked key groups, but they relied on these additional individuals for legitimacy and support. The largest network of policy champions was in Malawi in 2002, and this group was drawn from both researchers and policy makers, enabling them to very effectively bridge the research-to-policy gap. In Uganda, the policy champions were drawn from researchers and NGOs (CDC/Uganda, TASO and the MRC), actively recruiting other researchers and government policy makers to support the policy. In Zambia, policy champions eventually emerged from NGOs primarily funded by the United States government, representing only one of the three networks involved in policy making.

Additional file 3 summarises the key framework elements analysed here, and illustrates how they compare between our three country case studies as well as their interdependence.

## Discussion

The comparison of the country studies demonstrates how central a favourable healthcare context is to the adoption of research into policy. This is most visible in the case of the Malawi HIV Programme in 2002 and the Zambia HIV Programme in 2004/2005. In the former, the heavy emphasis on disease prevention through behaviour change, education and safe blood supplies left almost no room for a bio-medical approach, and a lack of interest in CPT (in contrast to the Malawi TB Programme which was searching for biomedical interventions). In Zambia (two years later), almost the reverse issue appeared, with a highly politicised bio-medical approach to HIV being dominated by a campaign to scale up ARVs, and the striking results of the research into CPT for HIV infected children were eclipsed. These policy processes show how an unfavourable policy context may make it difficult for policy development even when a sound evidence base upon which policy can be constructed exists.

Our analysis further illustrates how the evidence base is an important aspect of the policy development process, but that it does not in itself drive policy. In all cases, the policy development process for CPT began either with attempts to collect national data (Malawi) or with the publication of the trials in Cote d’Ivoire and the subsequent WHO/UNAIDS provisional recommendations for Africa (Uganda and Zambia). The strength of evidence was crucial: none of the governments in the three countries considered the WHO/UNAIDS provisional recommendations to be sufficiently evidence based to be implemented. In Malawi and Uganda, the coupling of this lack of evidence with the widespread perception of the usefulness of cotrimoxazole (and related drugs) as a treatment which might be threatened by its use as a prophylactic, was considered to be a powerful barrier to scale-up. Yet, this research also demonstrates clearly that while poor evidence may stall a policy process, even when powerful research evidence is available to policy makers, it does not necessarily get translated into policy: in our three countries, the only country to conduct randomized clinical trials took the longest amount of time to create national policy on CPT.

Finally, there appears to be a critical point when the policy implications of a piece of research evidence is contested (as was the case during the Malawian 2002 policy process), when a powerful policy entrepreneur, supported by policy champions can play a particularly important role in driving policy forward. A comparison of the two Malawian policy processes (in 2002 and 2005) is useful to demonstrate this as the same policy entrepreneur was involved in both processes but played very different roles. In 2002, he was highly active, and was supported by policy champions who defended the international research model when it was called into question and, with support from senior civil servants, drove the policy through. In contrast, in 2005 when there was more research evidence available which was almost universally accepted (the 2005 policy states that there was unanimous agreement) he played more of an administrative role, making sure that meetings took place, that the evidence was reviewed, and ultimately that the policy was written. Overall, the role of a policy entrepreneur was crucial in all cases, and, as an active agent, able to respond to and address barriers to policy development; providing additional evidence when questions were raised about the evidence base (particularly in Uganda and Malawi), making links between researcher and policy maker networks (most effectively in Malawi but also in Uganda and Zambia), and linking supportive policy champions with the evidence needed and the correct policy audience (in all three countries).

## Conclusion

The fact that national policy making on cotrimoxazole prophylaxis was varied, in terms of the content and time taken to reach a decision on its implementation in Malawi, Uganda and Zambia, demonstrates that national level variables must be considered to explain the uptake of evidence into policy settings. In our analysis the local context, interpretation of the evidence, and the nature of the links between policy makers and researchers were seen to both drive and stall the policy process. This three country study further reinforces the importance of understanding how these different facets of the policy process are interdependent. It shows the importance of a favourable policy context in which a policy entrepreneur can emerge; and the ways in which a well connected policy entrepreneur can act to overcome other obstacles, driving policy change when outstanding questions about the evidence may remain.

The development of the national CPT policy and guidelines across these countries reinforces the importance of analysing research-policy linkages with specific reference to country variables. As a retrospective analysis such as this, it enables us to better explain and understand the evidence to policy process. Yet this understanding can be used by policy making actors concerned with future use of evidence in policy as well, to help predict potential obstacles, or opportunities, for getting evidence into policy. Such prospective analyses are rare, but can be conducted by researchers and research funders early in the research cycle to ensure the right linkages with local representatives are in place for better evidence uptake once findings are available. The cross-country comparison has helped to validate and explore the usefulness of one specific framework, designed for developing country settings in particular. Improving the understanding of these processes can help to explain existing cases of the use (or non-use) of evidence in policy making, but can potentially also inform future strategies to bridge the research-policy gaps and improve the uptake of evidence in decision making.

## List of abbreviations used

WHO: World Health Organisation; UNAIDS: Joint United Nations Programme on HIV/AIDS; UNICEF: United Nations Children’s Fund; HIV: Human immunodeficiency virus; AIDS: Acquired immune deficiency syndrome

## Competing interests

The authors declare that they have no competing interests.

## Authors' contributions

EH participated in the design of the study, data collection, analysis and interpretation and drafted the paper. JP conceived of the study, participated in its design, supervised the analysis and interpretation of the data and supervised the drafting of the paper. SH conceived of the study, and participated in its design and coordination and helped to draft the manuscript. NC participated in the study design and coordination, data collection in Zambia and helped to draft the manuscript. SP participated in the study design and coordination, data collection in Malawi and helped to draft the manuscript. BD participated in the study design and coordination, data collection in Uganda and helped to draft the manuscript. DG conceived of the study, participated in its design, helped supervise the analysis and interpretation of the data. All authors read and approved the final manuscript.

## Supplementary Material

Additional file 1 Key events in policy and research Click here for file

Additional file 2 Variation in national policy content Click here for file

Additional file 3 Comparison of context, evidence and links in key policy processes across Malawi, Uganda and Zambia Click here for file
