# Supplementary material for: National policy development for cotrimoxazole prophylaxis in Malawi, Uganda and Zambia: the relationship between Context, Evidence and Links
Source: Health Res Policy Syst. 2011 Jun 16;9(Suppl 1):S6. doi: 10.1186/1478-4505-9-S1-S6 (PMC3121137; doi:10.1186/1478-4505-9-S1-S6)
Supplement: Additional file 1 — Key events in policy and research [file 1478-4505-9-S1-S6-S1.pdf]

Additional file 1 Key events in policy and research

Year	International	Malawi	Uganda	Zambia
1998	Anglaret et al present results of trial demonstrating efficacy of CPT for HIV related illness in Cote d'Ivoire at the XIIth World AIDS Conference, Geneva (Abstr LB 12464) [8].	ProTEST study begins examining feasibility of combining HIV and TB services.		
1999	Wiktor et al and Anglaret et al publish papers in the Lancet demonstrating efficacy of CPT for HIV related illness in Cote d'Ivoire at an early disease stage [7-8].	Boeree et al's trial on CPT for HIV infected TB patients begins [11].<br><br>Senior researchers at the College of Medicine write to the Lancet warning that Cote D'Ivoire studies may not be applicable to the whole of Africa [36].<br><br>UNAIDS stops funding Boeree et al's trial. The study is redesigned and continues [11].<br><br>Zacharia et al's study in Thyolo on the efficacy of CPT for HIV + TB patients begins [17].		
2000	WHO/UNAIDS publish provisional recommendations that CPT be provided to adults and children across Africa[29].	Mwaungulu et al's study on the efficacy of CPT for HIV+ TB patients begins [15].	Government rejects CDC/Uganda request to implement CPT in TASO clinics, asks for further data on its efficacy in Uganda.<br><br>Watera et al study into CPT begins [16].	Nunn et al's trial into CPT for HIV+ TB patients begins.
2001			Mermin et al study into CPT and safe water for HIV+ adults begins [13, 16].	Chintu et al's trial into CPT for HIV+ children begins.

2002		Van Oosterhout et al's study on incidence of severe events (hospitalizations and deaths) and clinical illness episodes among HIV infected adults that could potentially be prevented by CPT begins [35].<br><br>**POLICY CHANGE: Government adopts policy of CPT to HIV infected TB patients, based on results of local studies.**		
2003		Zacharia et al publish results demonstrating the efficacy of CPT for HIV infected TB patients [17].	Following dissemination meeting at the Ministry of Health, government allows for CPT to be implemented by TASO but rejects national scale up.	Chintu et al's trial ends early due to strength of results [5].
2004	WHO/UNAIDS/UNICEF release statement on the use of CPT for HIV exposed and infected children.	Mwaungulu et al publish results demonstrating the efficacy of CPT for HIV infected TB patients [15].	Mermin et al publish study results [13].	Chintu et al publish trial results [5].
2005		**POLICY CHANGE: Government adopts a policy of CPT for all HIV infected adults and children and publishes guidelines.**	**POLICY CHANGE: Government adopts a policy of CPT for HIV+ adults and children and publishes guidelines.**	
2006	WHO release detailed guidelines on CPT for HIV related disease in resource limited settings.			National scale up plan states commitment to providing CPT for HIV + patients.
2007				**POLICY CHANGE: The MOH release memo instructing use of CPT in HIV clinics**<br><br>National guidelines on CPT published.
