# Supplementary material for: National policy development for cotrimoxazole prophylaxis in Malawi, Uganda and Zambia: the relationship between Context, Evidence and Links
Source: Health Res Policy Syst. 2011 Jun 16;9(Suppl 1):S6. doi: 10.1186/1478-4505-9-S1-S6 (PMC3121137; doi:10.1186/1478-4505-9-S1-S6)
Supplement: Additional file 2 — Variation in national policy content [file 1478-4505-9-S1-S6-S2.pdf]

Additional file 2 Variation in national policy content

		National Guidelines for CPT			
Country		**Malawi**	**Malawi**	**Uganda**	**Zambia**
Year of publication		2002	2005	2005	2007
Infants	HIV exposed	None	From 6 weeks until HIV ruled out.		
	HIV infected	None	CPT regardless of clinical signs or symptoms, age or CD4 count.<br>Should not be discontinued.		<1yr- CPT regardless of symptoms and of CD4%<br>>1yr CPT if in stage 2, 3 or 4 or if CD4% <25%.<br>Should not be discontinued.
Children		2-5 year olds who are HIV infected with TB for the duration of the TB treatment. If patients wish to continue after this then they had to request CPT from local hospital.	CPT for all HIV infected children regardless of whether they are on ART.<br><br>Should not be discontinued.	CPT for all HIV infected children regardless of whether they are on ART.<br><br>Ideally for life. Discontinuation to be decided upon on an individual basis by the care provider.	1-5 year olds CPT if in stage 2, 3 or 4 or if CD4% <25%.<br><br><5 do not discontinue CTP.<br><br>>5 consider stopping CPT if good clinical and immune recovery, CD4 response, and secure supply of drugs.
Adults		All HIV infected TB patients for the duration of their treatment. If patients wish to continue after this then they had to request CPT from their local hospital.	CPT for adults in stages 2-4 or with CD4 count <500 cells/mm^3.<br><br>Should not be discontinued.	CPT to be given to all HIV infected adults.<br><br>Ideally for life. Discontinuation to be decided upon on an individual basis by the care provider.	CPT for all in WHO stages 2,3 and 4 regardless of CD4 count.<br><br>CD4 <350 cells/mm^3. regardless of clinical stage.<br><br>Discontinue CPT after CD4 >350 cells/mm^3. for >6 months<br>Monitor 3 monthly.
