# Supplementary material for: National policy development for cotrimoxazole prophylaxis in Malawi, Uganda and Zambia: the relationship between Context, Evidence and Links
Source: Health Res Policy Syst. 2011 Jun 16;9(Suppl 1):S6. doi: 10.1186/1478-4505-9-S1-S6 (PMC3121137; doi:10.1186/1478-4505-9-S1-S6)
Supplement: Additional file 3 — Comparison of context, evidence and links in key policy processes across Malawi, Uganda and Zambia [file 1478-4505-9-S1-S6-S3.pdf]

Additional file 3 Comparison of context, evidence and links in key policy processes across Malawi, Uganda and Zambia

Key policy process points	**Context**	**Evidence**	**Links**	**Policy Outcome**
**Malawi 2002**	TB Programme - Bio-medical approach with infrastructure to identify patients and provide drugs in the context of a well funded programme.<br><br>HIV Programme - *No bio-medical approach.*	Local, operational research showing efficacy for HIV+ TB patients but *no data on impact on cross resistance to similar drugs and no RCT.*<br><br>*No evidence on CPT in areas of high bacterial resistance.*	Dynamic policy entrepreneur located in TB programme with support from a group of senior policy champions who bridge the research/policy community. *No policy entrepreneur, champions, or networks identified linking to HIV programme.*	National CPT policy for HIV infected TB patients<br><br>*No policy for non-TB patients*
**Malawi 2005,**	Bio-medical approach within the HIV programme; Infrastructure being established to identify patients and provide drugs.	Solid evidence base of regional research both observational and RCT demonstrating efficacy of CPT. Local evidence showing the feasibility of CPT scale-up in the TB Programme.	Policy entrepreneur with policy champions which bridged the research/policy community.	National policy for all HIV infected children and adults
**Uganda 2005**	Early bio-medical approach of HIV programme, *but favouring ARVs.*<br>Strong donor agency and NGO partnership conducting research, with joint infrastructure in place to identify patients and provide drugs.	Local operational research showing efficacy for HIV+ patients with data on cross resistance. Solid evidence base of regional research both observational and RCT demonstrating efficacy of CPT.	Dynamic policy entrepreneur located in donor agency with support from a small group of policy champions mostly from donor agency/NGO partnership/research organisation partnership.	National policy for all HIV infected children and adults
**Zambia 2004/2005**	Bio-medical approach but with *strong political involvement seeking widespread access to ARVs in the public sector. Concerns about lack of infrastructure to deliver CPT.*	Local, randomised controlled trial demonstrating efficacy of CPT for HIV+ children.<br>*Interpreted as having clinical rather than policy relevance.*	*No policy entrepreneur identified.* Three policy champions identified - drawn from the research team and university physicians.	*No policy change*
**Zambia 2006/7**	Bio-medical approach with infrastructure to identify patients and provide drugs in the context of an existing ART programme.	Local, randomised trial demonstrating efficacy of CPT for HIV+ children. Solid regional evidence of observational research demonstrating efficacy of CPT for those HIV.	Strong policy entrepreneur with small group of senior policy champions drawn from NGOs undertaking programmatic work.	National policy for all HIV infected children and adults
